# Nursing knowledge of and attitude in cardiopulmonary arrest: cross-sectional survey analysis

**DOI:** 10.7717/peerj.6410

**Published:** 2019-02-07

**Authors:** Verónica Tíscar-González, Joan Blanco-Blanco, Montserrat Gea-Sánchez, Ascensión Rodriguez Molinuevo, Teresa Moreno-Casbas

**Affiliations:** 1 Nursing Teaching Supervision, OSI Araba, Osakidetza, Vitoria, Basque Country, Spain; 2 Faculty of Nursing and Physiotherapy, University of Lleida, Lleida, Spain; 3 Group for the Study of Society Health Education and Culture, GESEC, University of Lleida, Faculty of Nursing and Physiotherapy, Lleida, Spain; 4 Health Care Research Group, GRECS, Biomedical Research Institute of Lleida, Lleida, Spain; 5 Nursing Teaching and Research Unit, OSI Bilbao-Basurto, Osakidetza, Bilbao, Basque Country, Spain; 6 Nursing and Healthcare Research Unit, Institute of Health Carlos III, Madrid, Spain; 7 CIBERFES, Institute of Health Carlos III, Madrid, Spain

**Keywords:** Attitudes, Cardiopulmonary resuscitation, Ethics, Healthcare training, Knowledge, Nursing

## Abstract

**Background:**

Nurses are often the first to activate the chain of survival when a cardiorespiratory arrest happens. That is why it is crucial that they keep their knowledge and skills up-to-date and their attitudes to resuscitation are very important. The main aim of this study was to analyse whether the level of theoretical and practical understanding affected the attitudes of nursing staff.

**Methods:**

A questionnaire was designed using the Delphi technique (three rounds). The questionnaire was adjusted and it was piloted on a test-retest basis with a convenience sample of 30 registered nurses. The psychometric characteristics were evaluated using a sample of 347 nurses using Cronbach’s alpha. Descriptive analysis was performed to describe the sociodemographic variables and Spearman’s correlation coefficient to assess the relationship between two scale variables. Pearson’s chi-squared test has been used to study the relationship between two categorical variables. Wilcoxon Mann Whitney test and the Kruskal–Wallis test were performed to establish relationships between the demographic/work related characteristics and the level of understanding.

**Results:**

The Knowledge and Attitude of Nurses in the Event of a Cardiorespiratory Arrest (CAEPCR) questionnaire comprised three sections: sociodemographic information, theoretical and practical understanding, and attitudes of ethical issues. Cronbach’s alpha for the internal consistency of the attitudes questionnaire was 0.621. The knowledge that nurses self-reported with regard to cardiopulmonary arrest directly affected their attitudes. Their responses raised a number of bioethical issues.

**Conclusions:**

CAEPCR questionnaire is the first one which successfully linked knowledge of cardiopulmonary resuscitation to the attitudes towards ethical issues Health policies should ensure that CPR training is mandatory for nurses and all healthcare workers, and this training should include the ethical aspects.

## Introduction

Early initiation of cardiopulmonary resuscitation (CPR) manoeuvres and activation of the chain of survival are key factors in the prognosis of patients who have suffered a cardiorespiratory arrest (CRA) (Balcázar-Rincón, Mendoza-Solís & Ramirez-Alcántara, 2015). Therefore, it is important to understand and master CPR techniques, as these are the main determinants of success rates in CRA care, irrespective of the setting in which they are performed.

Early initiation of CPR manoeuvres is so important that over the past few years training efforts have targeted the general public, especially in primary and secondary schools (López-Messa et al., 2011; Hansen et al., 2017; Sorets & Mateen, 2015). This training has showed them how activate the chain of survival and initiate CPR manoeuvres as soon as possible.

However, internationally some studies have reported a lack of knowledge among healthcare professionals, including nurses (Muñoz Camargo et al., 2011; Almeida et al., 2011; Medina-Hernando & Martinez-Ávila, 2013; Plagisou et al., 2015) and physicians (Balcázar-Rincón, Mendoza-Solís & Ramirez-Alcántara, 2015; Howell et al., 2014). Basic aspects of CPR, such as the correct compression and ventilation sequence, do not appear to be correctly understood by all healthcare professionals and it has been suggested that CPR training needs to be improved in medical (López-Messa et al., 2011) and nursing schools (López-Messa et al., 2011; Medina-Hernando & Martinez-Ávila, 2013; Dal & Sarpkaya, 2013), as well as postgraduate training (Medina-Hernando & Martinez-Ávila, 2013).

In 2010 the International Liaison Committee on Resuscitation (ILCOR) reported that basic and advanced life-support knowledge and skills are likely to deteriorate over a short period of time, approximately 3–6 months (Dal & Sarpkaya, 2013; Mokhtari Nori et al., 2012; Nolan et al., 2010), and it recommends periodic evaluations to identify those professionals who need to refresh their knowledge and/or skills (Nolan et al., 2010).

The reported incidence of intra-hospital cardiopulmonary arrest in Spain differs widely between, from one to five patients per 1,000 patients admitted, with an overall survival rate of 20% (Sandroni et al., 2007).

Nurses are generally the first healthcare professionals to detect CRA and activate the chain of survival at healthcare institutions (Nyman & Sihvonen, 2000) and it is crucial that they keep their knowledge and skills updated (Dal & Sarpkaya, 2013).

Deciding whether to initiate and/or stop CPR manoeuvres is sometimes difficult, because ethical and legal issues may influence the decision-making process (Pyl & Menard, 2012; Püttgen & Geocadin, 2007). Over the past 10 or 20 years most elderly patients institutionalized in the USA have died with a written limited therapeutic effort (LTE) order (Cherniack, 2002). Similarly, LTEs and the interest in ethical issues in clinical practice have also increased in other countries (Bueno Muñoz, 2013). Do not Resuscitate (DNR) decisions are part of LTE, and they can be affected by factors as functional and premorbid status, quality of life and probability of survival. A systematic review conducted by Cook (Cook et al., 2017), suggested that it could be ageism in some DNR decisions when the decisions don’t take in account other factors like the described before. International and local cultural, legal, religious and socioeconomic factors are very important in the decision making too (Lippert et al., 2010).

Written LTEs are not common in Spain (Monzón et al., 2010) and it has been reported that the trend is to practice defensive medicine. This can provide care to CRA patients that may be often considered excessive (Santana Cabrera et al., 2010; Alonso-Renedo, González-Ercilla & Iráizoz-Apezteguía, 2014).

Some studies suggest that health professionals show some controversy to the presence of relatives during the CPR manoeuvres (Tíscar-González et al., 2018; Colbert & Adler, 2013; Enriquez et al., 2017), even when the literature reflects that it could be positive for the family to improve the grieving process (Oczkowski et al., 2015; Moons & Norekvål, 2008).

For all this, the attitudes displayed by nurses in the event of CRA are essential, as these determine whether they will activate the chain of survival as early as possible, when required, and also to safeguard the patient’s right to receive dignified treatment that respects fundamental ethical principles.

But how are these attitudes reflected in clinical practice? The conceptual construct of the term attitude is complex. It may be defined as a tendency or predisposition, with cognitive, behavioural and, above all, positive and negative emotional components towards a certain situation. The measurement and evaluation of attitudes have been studied within the framework of social psychology for many years and have proved to be complicated (Manassero Mas & Vazquez Alonso, 2001; Vázquez-Alonso & Manassero-Mas, 1997).

The main aim of this study was to analyse whether the level of theoretical and practical understanding that nursing staff have is reflected in their attitudes to bioethical issues.

No validated questionnaires were available that evaluated the theoretical and practical understanding of CPR, and the attitudes exhibited by professionals in the event of CRA. Therefore, other aim of this study was to develop a questionnaire to measure how well nurses understood CPR and their attitudes to the bioethical issues surrounding CRAs.

## Material & Methods

A literature review and a triangulation between the research team and an expert in questionnaires validation were performed in order to create the CAEPCR questionnaire. One *ad hoc*, self-administered questionnaire was designed, which was based on the 2010 Basic and Advanced CPR recommendations from the European Resuscitation Council (Lippert et al., 2010) and current ethical and legal recommendations. It included both social and work-related characteristics**,** knowledge questionnaire and attitudes questionnaire.

The result was submitted to a further 10 experts for evaluation using the Delphi technique (Landeta, 2006; Yañez & Cuadra, 2008). These experts were carefully selected and included physicians and nurses from different care levels and geographic regions, namely the Basque Country and Catalonia. The selection criteria were based on their clinical experience in hospital-based, primary and intensive care, basic and advanced CPR-related teaching experience and/or advanced understanding of research methodology and questionnaire validation. Contact was via email and a total of three rounds of feedback were conducted.

**Figure 1:**
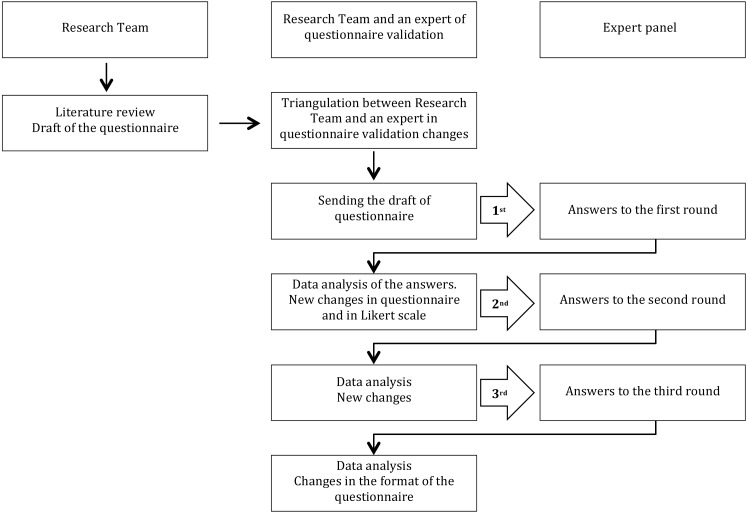
Delphi technique flow chart.

With the Delphi technique, the questionnaire is subjected to the evaluation of experts, who must assess the latter’s ability to evaluate all the dimensions that we want to measure. This technique presupposes that. Although there may be a discrepancy, some points can be found of consensus, and differentiate which aspects or dimensions are important (Fig. 1).

A test-retest pilot study was carried out after applying the Delphi technique to the questionnaire and performing the required modifications. A convenience sample of 30 registered nurses from the main hospital and outpatient departments at the OSI Bilbao-Basurto Integrated Healthcare Organisation was used. First, the psychometric characteristics of the questionnaire were measured: reliability using the intra-class correlation coefficient, internal consistency using Cronbach’s alpha. The attitudes section of the questionnaire was subsequently modified. The Spanish questionnaire comprised 31 items: the first section covered Social/work related data, the second section theoretical and practical understanding and the third section covered attitudes towards ethical aspects (Tíscar González et al., 2015).

The nurses’ theoretical and practical understanding was evaluated using 11 questions that each had one correct and three incorrect answers. Similarly, attitudes toward ethical aspects were assessed by presenting 12 statements that were accompanied by a five-point Likert-type scale ranging from one for strongly disagree to five for strongly agree.

Last, a cross-sectional design study was performed. A total of 2,180 registered nurses were invited to participate. As inclusion criteria they should be working in hospitals and primary care organizations during the data collection period (from 16 October to 9 June 2015). The questionnaire was made available to the target population online using the Google doc tool and a link was sent to their corporate email accounts. The link was sent twice, two weeks apart. The body of the message comprised a poster containing images and encouraging and challenging professionals to participate.

### Statistical method

Statistical analysis: a Microsoft Excel database was constructed for the statistical treatment and analysis using the SPSS 21 statistics programme. A *p* value of less than 0.05 was considered statistically significant, unless de Bonferroni correction was applied (*p* value < 0.005).

Descriptive analysis was performed to describe the sociodemographic variables and Spearman’s correlation coefficient to assess the relationship between two scale variables. Pearson’s chi-squared test has been used to study the relationship between two categorical variables, using Bonferroni correction for *p* value < 0.05. Wilcoxon Mann Whitney test and the Kruskal–Wallis test were performed to establish relationships between the demographic/work related characteristics and the level of understanding. When The psychometric characteristics of the questionnaire and their internal consistency were evaluated using Cronbach’s alpha (Yañez & Cuadra, 2008). Finally, twelve attitude questions were analysed using Principal Components Analysis (PCA).

### Ethical considerations

Approval was obtained from the Clinical Research Ethics Committee of OSI Bilbao-Basurto (Osakidetza). The study adhered to the rules of the Declaration of Helsinki.

## Results

The CAEPCR questionnaire was created and modified according to the three Delphi rounds (version 0) and the results of the pilot study (version 1).

347 (15.9%) of the 2,180 nurses who were invited to participate in the study completed the questionnaire. The response rate was similar to that reported by other studies developed in our country (Aerny Perreten et al., 2012; Lupiáñez-villanueva, 2008).

### Social/work-related characteristics

The social/work related characteristics are presented in Table 1.

**Table 1:** Demographics/work related characteristics.

**Demographics/work related characteristics** (*N* = 347)	
**Demographics**	
**Age** (mean; SD)	40.28; 10.65
**Sex Female *n* (%)**	302 (87)
**Social/work related characteristics**	
**Work experience**	
<5 years	50 (14.4)
>20 years	122 (35.2)
11–15 years	64 (18.4)
16–20 years	38 (11.0)
5–10 years	73 (21.0)
**Type of contract**	
Eventual contract	164 (47.3)
Permanent contract	183 (52.7)
**Service**	
Consultation Paediatrics Primary Care	13 (3.7)
Primary Care Consultation	102 (29.4)
Paediatric hospital service	9 (2.6)
Medical unit	157 (45.2)
Surgical Unit	66 (19)
**Last CPR recycling course**	
<6 months	22 (6.3)
>2 years	174 (50.1)
1–2 years	79 (22.8)
6 m–1 year	33 (9.5)
Never	39 (11.2)
**Last CPR attended**	
<6 months	41 (11.8)
>2 years	157 (45.2)
1–2 years	34 (9.8)
6 m–1 year	39 (11.2)
Never	76 (21.9)

We found that 87.0% (*n* = 302) of the 347 participants were female, their median age was 40 years and 35.2% (*n* = 122) had worked as nurses for more than 20 years. With regard to their employment status, 52.7% (183) had a permanent contract and 47.3% (164) were working on a temporary or provisional basis.

We found that 50.1% (*n* = 174) of nurses said that more than two years had passed since they last received CPR-related training, 22.8% (*n* = 79) had received training in the last 1–2 years, 9.5% (*n* = 33) in the 6-12 months, 6.4% (*n* = 22) in the last six months and 11.2% (*n* = 39) had never received CPR-related training.

The findings also showed that 21.9% (*n* = 76) of the nurses had never participated in CPR manoeuvres and 45.2% (*n* = 157) had not attended a CRA in the past two years.

When it came to refresher courses, 13.5% (*n* = 47) felt that they should be held every six months, 51.0% (*n* = 177) every 6-12 months and 33.1% (*n* = 115) every 1-2 years.

#### Theoretical/practical understanding of CPR

With regard to the theoretical/practical understanding of CPR recommendations, a mean number of 7.1 ±1.45 correct answers were obtained for the 11 items on the questionnaire.

The frequency of correct answers in theoretical and practical knowledge section of the questionnaire are presented in Table 2.

**Table 2:** Frequency of correct answers in the theoretical/practical knowledge sections of the questionnaire.

Item	Correct answer *n* (%)	
Q1. A family member tells you that a patient is lying on the floor and is not responding, you therefore	270 (77.8)	
Q2. You have checked that the patient is actually unconscious, therefore you now	186 (53.6)	
Q3. After checking for the absence of breathing and a pulse, you reach the conclusion that the patient is in cardiorespiratory arrest (CRA), therefore you start CPR manoeuvres. Consequently, you must know the thoracic compression technique	243 (70)	
Q4. According to the ERC 2010 recommendations, the correct compression/ventilation relationship in CPR is	347 (100)	
Q5. The most important changes that have been made in the updates to the CPR guidelines include	330 (95.1)	
Q6. During treatment of ventricular fibrillation-related cardiac arrest or pulseless ventricular tachycardia (PVT), 1 mg of adrenaline is administered	82 (23.6)	
Q7. For thoracic compression to be of good quality, whenever possible you are recommended to	225 (64.8)	
Q8. After performing defibrillation you must	36 (10.4)	
Q9. With regard to the administration route for medicines in CRA, it is true that	311 (89.6)	
Q10. The drugs used intravenously must	102 (29.4)	
Q11. What is the most effective treatment for a victim with ventricular fibrillation-related CRA	315 (90.8)	

Statistically significant differences were found for two items—questions seven and 11—with regard to the association between the number of correct answers and how long the participants had worked as nurses. Nurses with more than 16 years of experience reported the lowest frequency of correct answers (*p* = 0.02) to question seven, which covered what they were recommended to do to achieve good-quality thoracic compression. Nurses on permanent contracts gave the highest number of correct answers (*p* = 0.02) to question 11 about the most effective treatment for a victim with ventricular fibrillation-related CRA (Table 3). Applying Bonferroni corrections for multiple comparting these significant differences disappear.

**Table 3:** Frequency of correct answers in the theoretical/practical knowledge sections of the questionnaire by work experience and type of contract.

		Work experience *n* (% of correct answers)						Type of contract *n* (% of correct answers)		
Item	*n* (%)	<5 years	5–10 years	11–15 years	16–20 years	>20 years	*p*-Value	Temporary	Permanent	*p*-Value
Q1.	270 (77.8)	38 (76)	61 (83.6)	51 (79.7)	31 (81.6)	89 (73)	0.46	129 (78.7)	141 (77)	0.72
Q2.	186 (53.6)	33 (66)	41 (56.2)	31 (48.4)	21 (55.3)	60 (49.2)	0.29	96 (58.5)	90 (49.2)	0.08
Q3.	243 (70)	36 (72)	55 (75.3)	49 (76.6)	28 (73.7)	75 (61.5)	0.14	117 (71.3)	126 (68.9)	0.61
Q4.	347 (100)	50 (100)	73 (100)	64 (100)	38 (100)	122 (100)	1	164 (100)	183 (100)	1
Q5.	330 (95.1)	47 (94)	70 (95.9)	62 (96.9)	36 (94.7)	115 (94.3)	0.93	154 (93.9)	176 (96.2)	0.33
Q6.	82 (23.6)	14 (28)	19 (26)	13 (20.3)	11 (28.9)	25 (20.5)	0.66	38 (23.2)	44 (24)	0.85
Q7.	225 (64.8)	38 (76)	51 (69.9)	47 (73.4)	20 (52.6)	69 (56.6)	0.02^a^	114 (69.5)	111 (60.7)	0.08
Q8.	36 (10.4)	6 (12)	9 (12.3)	6 (9.4)	4 (10.5)	11 (9)	0.94	19 (11.6)	17 (9.3)	0.48
Q9.	311 (89.6)	45 (90)	65 (89)	60 (93.8)	34 (89.5)	107 (87.7)	0.79	144 (87.8)	167 (91.3)	0.29
Q10.	102 (29.4)	11 (22)	21 (28.8)	22 (34.4)	16 (42.1)	32 (26.2)	0.22	45 (27.4)	57 (31.1)	0.45
Q11.	315 (90.8)	43 (86)	64 (87.7)	59 (92.2)	31 (81.6)	118 (96.7)	0.02^a^	139 (84.8)	176 (96.2)	0.02
		Mean (SD)					
Final evaluation	6.4 (1.3)	6.6 (1.4)	6.6 (1.2)	6.6 (1.1)	6.5 (1.4)	6.1 (1.3)	0.07	6.4 (1.3)	6.4 (1.2)	0.85

**Notes.**

a Any comparison is significant after applying Bonferroni correction (*p*-Value < 0.005).

The overall number of correct answers, with a mean of 6.41 ± 1.32 out of 10, showed a statistically significant negative relationship with the age of the professional (*r* =  − 0.18, *p* = 0.01). Nurses aged more than 46 years had the lowest scores and these were particular lower when the respondents were 56 years or more. The mean scores did not differ significantly in terms of the type of contract (*p* = 0.85) (Table 3), how long they had worked as a nurse (*p* = 0.07) (Table 3), or where the nurse was working (*p* = 0.47) (Table 4).

**Table 4:** Frequency of correct answers in the theoretical/practical knowledge sections of the questionnaire by service.

	Service *n* (% of correct answers)					
Item	Medical unit	PC consultancy	Surgical unit	Paediatric PC consultancy	Paediatric service	*p*-Value
Q1.	120 (76.4)	82 (80.4)	53 (80.3)	7 (53.8)	8 (88.9)	0.22
Q2.	83 (52.9)	53 (52)	38 (57.6)	6 (46.2)	6 (66.7)	0.83
Q3.	112 (71.3)	68 (66.7)	48 (72.7)	8 (61.5)	7 (77.8)	0.81
Q4.	157 (100)	102 (100)	66 (100)	13 (100)	9 (100)	
Q5.	149 (94.9)	98 (96.1)	61 (92.4)	13 (100)	9 (100)	0.67
Q6.	33 (21)	26 (25.5)	17 (25.8)	2 (15.4)	4 (44.4)	0.46
Q7.	113 (72)	59 (57.8)	45 (68.2)	6 (46.2)	2 (22.2)	0.01^a^
Q8.	17 (10.8)	12 (11.8)	6 (9.1)	1 (7.7)	0 (0)	0.82
Q9.	142 (90.4)	91 (89.2)	57 (86.4)	13 (100)	8 (88.9)	0.66
Q10.	57 (36.3)	19 (18.6)	17 (25.8)	4 (30.8)	5 (55.6)	0.01^a^
Q11.	139 (88.5)	93 (91.2)	62 (93.9)	13 (100)	8 (88.9)	0.54
	
Final evaluation mean (SD)	6.5 (1.4)	6.3 (1.1)	6.5 (1.2)	6 (1.5)	6.7 (1.8)	0.47

**Notes.**

a Any comparison is significant after applying Bonferroni correction (*p*-Value < 0.005).

The nurses who had undertaken a refresher course less than one year previously, and those who had attended a CRA within the past six months, obtained a higher understanding score. However, the differences were not statistically significant when they were compared with the nurses who had not had this training (*p* = 0.35 and *p* = 0.09 respectively) (Table 5).

**Table 5:** Evaluation of knowledge in terms of attendance at CPR refresher course, last time CRA attended and need for CPR training.

	Evaluation of the knowledge questionnaire—mean (SD)					
	<6 months	6–12 months	1–2 years	>2 years	Never	*p*-Value
Time since last CPR refresher course	6.7 (1.2)	6.7 (1.2)	6.3 (1.2)	6.4 (1.3)	6.3 (1.3)	0.35
Time since last attended a CRA	6.8 (1.4)	6.5 (1.3)	6.6 (1)	6.4 (1.3)	6.1 (1.3)	0.09
Need for CPR training course	6.4 (1.2)	6.5 (1.3)	6.3 (1.3)	5.9 (1.1)	–	0.23

### Attitude questionnaire

#### Psychometric characteristics

The internal consistency of the 12-item attitudes section of the questionnaire had a Cronbach’s alpha value of 0.62. To study the reduction of scales, a principal components factor analysis was developed, which led to four components (factors).

The common factors and component matrix of the principal component analysis are summarized in Table 6.

**Table 6:** Rotated component matrix.

Attitude questions	Component			
	1	2	3	4
1: Do you consider yourself to be sufficiently well trained to perform CPR?	0.90	0.01	0.09	−0.06
2: Do you consider that you understand the action protocol for performing CPR in your work area?	0.90	0.01	0.05	−0.01
3: Do you consider yourself to be personally responsible for being able to perform CPR?	0.46	0.10	−0.14	0.25
4: Do you consider it to be the responsibility of your work centre to provide you with training to perform CPR?	0.22	0.30	−0.18	0.46
5: Do you consider that the person with the greatest understanding and experience in the team should be the person to LEAD CPR irrespective of whether they are a physician or a nurse?	−0.13	0.72	0.13	0.15
6: Do you consider that CPR can be PERFORMED by either physicians or nurses?	0.10	0.79	0.01	0.12
7: Do you agree that you do not need to be a healthcare professional to initiate CPR?	0.09	0.56	0.01	0.12
8: Do you consider it appropriate not to start, or to interrupt CPR manoeuvres if started, when the probability of neurological sequel is high?	−0.03	0.06	0.72	0.09
9: Do you consider that the presence of family members does not influence your decision to commence CPR manoeuvres?	0.09	0.44	0.37	−0.30
10: Do you believe that the information YOU have about the patient may lead you to stop CPR?	0.03	0.03	0.79	0.17
11: Do you consider it necessary to identify do not resuscitate patients (for example in the hospital or even in the primary care records)?	0.09	0.22	0.25	0.63
12: Do you consider it necessary for patients at highest risk of requiring CPR to be identified in the hospital?	−0.07	0.02	0.20	0.74

Factor 1 was named Personal Responsibility and included three items (1, 2 and 3). Cronbach’s alpha value was 0,66.

Factor 2, Leadership and decision making, included four items (5, 6, 7 and 9). Cronbach’s alpha value of 0.51.

Factor 3, Stop CPR, included two items (8 and 9). Cronbach’s alpha value of 0.53.Factor 4, Organization Responsibility, included three items (4, 11 and 12). Cronbach’s alpha value of 0.44.

### Outcomes of Attitudes questionnaire

4% (*n* = 14) of nurses strongly agreed and 20% (*n* = 70) slightly agreed with the statement that they were sufficiently well prepared to perform CPR at their place of work, while 4% (*n* = 15) strongly agreed and 27% (*n* = 93) slightly agreed to some extent that they were aware of the action protocol at their centre.

37% (*n* = 129) strongly agreed and 25% (*n* = 87) slightly agreed that staff were responsible for instruction to attend a CRA while 75% (261) strongly agreed and 18% (63) slightly agreed that their place of work was responsible for providing CPR-related training.

We found that 73.5% (*n* = 255) of nurses strongly agreed that the person with the greatest knowledge and experience in the team, should lead the CPR and that CPR could be performed by either physicians or nurses (67.7%, *n* = 235).

23.1% (*n* = 80) strongly agreed and 21.6% (*n* = 75) slightly agreed that not to start or to interrupt CPR manoeuvres if started, when the probability of neurological sequelae is high.

27.1% (*n* = 94) strongly agreed and 21.6% (*n* = 75) slightly agreed that the presence of relatives members does not influence their decision to commence CPR manoeuvres.

22.2% (*n* = 77) strongly agreed and 26.8% (*n* = 93) slightly agreed that the information they have about the patient may lead them to stop CPR.

78.1% (*n* = 271) of nurses strongly agreed and 14.1% (*n* = 49) slightly agreed that DNR patients should be identified in their clinical records.

The years of experience that a nurse had did not have any impact on the attitude items, except for the item that asked them if the presence of family members influenced their decision to commence CPR (*p* = 0.04). The percentage of nurses who agreed or strongly agreed increased with the number of years they had spent as a nurse: 42% (*n* = 21∕50) with less than five years’ experience and 61.5% (*n* = 75∕122) with more than 20 years’ experience. The type of nursing contract also affected item nine, that asked whether the presence of family members influenced their decision to start CPR manoeuvres (*p* = 0.01), with the percentage who agreed or strongly agreed being higher for permanent than temporary staff at 59.6% (*n* = 109∕183) versus 46.9% (*n* = 77∕164). A statistically significant relationship was also found for the age of the nurse and the item relating to the presence of family members (*p* = 0.01), with a Spearman’s rho of 0.16 reflecting a higher degree of agreement in older nurses.

The degree of agreement for four items exhibited significant differences with regard to the time since the nurses attended their last CPR refresher course. These related to being sufficiently well trained to perform CPR (item 1, *p* = 0.01), understanding the action protocol for CPR in their work area (item 2, *p* < 0.01) whether they though CPR could be performed by either nurses of physicians (item 6, *p* = 0.01) and whether it was appropriate to start CPR if the probability of neurological sequelae was high (item 8, *p* = 0.01). The highest percentage of nurses who agreed or strongly agreed were those who had gone the longest without attending such a course (Table 7). After the Bonferroni corrections the significant differences are between less than 6 months and more than 2 years or never. With regard to the time since they had last attended a CRA, statistically significant differences were found for items 1 and 2 (*p* < 0.01) and 10 (*p* = 0.02), which asked if information they had about the patient would lead then to stop CPR. Nurses who had attended a CRA course most recently exhibited higher levels of agreement (Table 7). The significance different appear between less than 6 months and more than 2 years or never. With regard to the relationship between the mean score in the theoretical and practical sections of the questionnaire and the attitudes exhibited by the nurses in the event of a CRA, a significant association was found between the score and the degree to which they felt prepared to perform CPR (item 1) (rho 0.22; *p* < 0.01), with higher scores showing a higher degree of agreement. A similar relationship was found with understanding the CRA action protocol of their organization (item 2), with higher scores showing a higher degree of agreement (rho 0.21; *p* < 0.01). A higher degree of agreement was found between someone who was not a healthcare professional starting CPR (item 7) and a higher score in the understanding section of the questionnaire (rho 0.15; *p* = 0.01). The degree of agreement with not starting or stopping CPR manoeuvres when the probability of neurological sequelae was high (item 8) increased with a higher understanding score (rho 0.15; *p* = 0.01). The degree of agreement that the information that nurses had about the patient may lead them to stop CPR (item 10) increased with a higher understanding score (rho 0.18; *p* = 0.01).

## Discussion

This study shows how theoretical and practical understanding of nurses with regard to CRA directly affected the attitudes towards ethical issues that they exhibited in that context. Some studies, as developed by Källestedt (Källestedt et al., 2012) in Sweden, showed that CPR education influenced the general attitudes, as feelings (secure, anxious…) of healthcare professionals. However, there is gap in the scientific literature about healthcare professionals’ attitudes towards ethical issues in the CPR, so we can’t compare our outcomes with other authors.

In relation to the Knowledge questionnaire complexity the wide range of correct answers in Knowledge questionnaire −10.4% to 100%—suggests that some of the questions were more difficult than others.

**Table 7:** Slighty agree and strongly agree frequency in outcomes from the attitudes questionnaire.

Item	Slightly agree and strongly agree *n* (%)					
	<6 months	6–12 months	1–2 years	>2 years	Never	*p*-Value
	**Time since last CPR refresher course**					
Attitude 1	10 (45.4%)	12 (36.4%)	18 (22.8%)	39 (22.4%)	5 (12.8%)	0.01^a^
Attitude 2	14 (63.7%)	15 (45.4%)	28 (35.5%)	44 (25.3%)	7 (17.9%)	<0.01^b^
Attitude 3	14 (63.7%)	22 (66.6%)	56 (70.9%)	104 (59.7%)	20 (51.2%)	0.05
Attitude 4	22 (100%)	32 (97%)	76 (96.2%)	161 (92.5%)	33 (84.6%)	0.14
Attitude 5	22 (100%)	30 (91%)	68 (86.1%)	155 (89.1%)	37 (94.8%)	0.79
Attitude 6	22 (100%)	28 (84.9%)	69 (87.3%)	151 (86.8%)	38 (97.4%)	0.03
Attitude 7	21 (95.5%)	30 (90.9%)	61 (77.2%)	130 (74.7%)	31 (79.5%)	0.11
Attitude 8	9 (40.9%)	14 (42.4%)	31 (39.3%)	82 (47.2%)	19 (48.7%)	0.00^a^
Attitude 9	13 (59.1%)	16 (48.5%)	41 (51.9%)	95 (54.6%)	21 (53.8%)	0.43
Attitude 10	12 (54.6%)	12 (36.3%)	40 (50.7%)	84 (48.3%)	22 (56.4%)	0.37
Attitude 11	21 (95.5%)	33 (100%)	70 (88.6%)	161 (92.5%)	35 (89.7%)	0.29
Attitude 12	13 (59.1%)	28 (84.9%)	63 (79.7%)	130 (74.7%)	31 (79.5%)	0.62
	**Time since last attended a CRA**					
Attitude 1	20 (48.7%)	10 (25.6%)	10 (29.4%)	34 (21.6%)	10 (13.2%)	<0.01^c^
Attitude 2	25 (61%)	16 (41%)	16 (47%)	38 (24.2%)	13 (17.1%)	<0.01^d^
Attitude 3	25 (61%)	21 (53.9%)	26 (76.5%)	98 (62.4%)	46 (60.5%)	0.09
Attitude 4	40 (97.5%)	36 (92.3%)	33 (97.1%)	146 (93%)	69 (90.8%)	0.26
Attitude 5	36 (87.8%)	30 (77%)	29 (85.3%)	147 (93.6%)	70 (92.1%)	0.28
Attitude 6	38 (92.7%)	32 (82%)	31 (91.1%)	140 (89.2%)	67 (88.2%)	0.61
Attitude 7	32 (78%)	25 (64.1%)	25 (73.5%)	134 (85.4%)	57 (75%)	0.13
Attitude 8	21 (51.2%)	13 (33.3%)	14 (41.1%)	80 (51%)	27 (35.6%)	0.08
Attitude 9	24 (58.5%)	18 (46.1%)	20 (58.8%)	85 (54.2%)	39 (51.3%)	0.05
Attitude 10	23 (56.1%)	25 (64.1%)	13 (38.3%)	84 (53.5%)	25 (32.9%)	0.02
Attitude 11	37 (90.2%)	34 (87.2%)	34 (100%)	149 (94.9%)	66 (86.9%)	0.12
Attitude 12	26 (63.4%)	25 (64.1%)	30 (88.2%)	124 (79%)	60 (79%)	0.41
	**Need for CPR training course**					
Attitude 1	10 (21.2%)	46 (26%)	26 (22.6%)	2 (25%)	–	0.24
Attitude 2	17 (36.2%)	56 (31.6%)	33 (28.6%)	2 (25%)	–	0.47
Attitude 3	23 (49%)	116 (65.6%)	71 (61.8%)	6 (75%)	–	0.05
Attitude 4	41 (87.2%)	169 (95.4%)	108 (93.9%)	6 (75%)	–	0.07
Attitude 5	42 (89.4%)	159 (89.9%)	103 (89.6%)	8 (100%)	–	0.83
Attitude 6	41 (87.2%)	159 (89.8%)	100 (87%)	8 (100%)	–	0.93
Attitude 7	37 (78.8%)	137 (77.4%)	93 (80.8%)	6 (75%)	–	0.93
Attitude 8	23 (48.9%)	73 (41.2%)	56 (48.7%)	3 (37.5%)	–	0.69
Attitude 9	23 (49%)	91 (51.4%)	66 (57.4%)	6 (75%)	–	0.81
Attitude 10	25 (53.1%)	83 (46.9%)	58 (50.5%)	4 (50%)	–	0.65
Attitude 11	44 (93.6%)	162 (91.6%)	108 (93.9%)	6 (75%)	–	0.08
Attitude 12	38 (80.9%)	134 (75.7%)	88 (76.5%)	5 (62.5%)	–	0.09

**Notes.**

a Any comparison is significant after applying Bonferroni correction (*p*-Value <  0.005).

b Comparison between <6 months and >2 years (*p*-Value = 0.001) and between <6 months and never (*p*-Value = 0.001) are significant after applying Bonferroni correction.

c Comparison between <6 months and >2 years (*p*-Value = 0.001) and between <6 months and never (*p*-Value <  0.0001) are significant after applying Bonferroni correction.

d Comparison between <6 months and >2 years (*p*-Value <  0.0001) and between <6 months and never (*p*-Value <  0.0001) are significant after applying Bonferroni correction.

The registered nurses who participated in the study achieved a mean qualification of 7.1 out of 11 in the theoretical and practical knowledge section of the questionnaire on CPR. This gap shows considerable room for improvement (Muñoz Camargo et al., 2011; Almeida et al., 2011; Medina-Hernando & Martinez-Ávila, 2013). Most of the professionals who participated in this study didn’t feel enough prepared to perform CPR which coincide with Medina’s outcomes (Medina-Hernando & Martinez-Ávila, 2013).

In our study, 50% of the study nurses had not received CPR related training for more than two years, which contrasted with a study by Dal and Sarpkaya (Dal & Sarpkaya, 2013), who recommended that theoretical and skills training should be ideally be repeated every six months.

In other countries, such as the USA, health professionals need up-to-date CPR certification, namely a CPR card, to carry out clinical work (Sorets & Mateen, 2015). The USA and several European countries provide CPR instruction in secondary schools (Hansen et al., 2017; Semeraro et al., 2016). There are no legal requirements in our country and that could explain why 50% of the nurses in our study sample had not received CPR training in the last two years.

Moreover, those nurses who had undertaken a refresher course within the last year and those who had attended a CRA course within the past six months recorded higher understanding scores, which suggest that updated training is useful.

It should be stressed that the majority of nurses (93%) considered that the health institution where they worked should provide and maintain CPR training.

On the other hand, there was widespread agreement that those patients who were not candidates for starting CPR manoeuvres should be identified in their clinical records. Most of nurses participating in this study considered that it is necessary to identify DNR patients in their history records. Spanish health organisations do not currently use DNR orders, soa comprehensive document covering patients’ wishes should be considered by all hospitals and primary care organisations (Monzón et al., 2010). This situation contrasts with the outcomes reported in other countries. A study developed by  Mukamel et al. (2013) in the USA showed that the 40–50% of patients in a long term nursing home had a DNR order, and in Canada almost 70% of patients had DNR order (Brink, 2014).

Healthcare professionals must be concerned by the ethical issues that may surround CRA and consider the potential impact of their attitudes towards patient survival and quality of life (Hayes, 2013).

As repercussions for clinical practice we highlight that the CAEPCR questionnaire could be useful in the design of empirical mechanisms to improve and adapt both undergraduate and postgraduate training to the knowledge and attitude needs of nurses, in order to improve the quality of CPR manoeuvres. In addition, it could be useful to evaluate the attitudes of other professionals involved in CPR manoeuvres, although in the context of our study we have only addressed nurse vision. This can be expected to improve the prognosis of patients by preventing dysthanasia in cases where the patient cannot benefit from CPR.

One limitation of the study was Cronbach’s alpha for the attitudes questionnaire, which did not reach the ideal value of 0.70 (Ponterotto & Ruckdeschel, 2007; Campbell, 1976; Portney & Watkins, 1993). According to authors such as (Huh, Delorme & Reid, 2006), the reliability value in an exploratory study should be above 0.6, reaching a minimum of 0.7 in non-explorative studies, thus reaching the necessary level of reliability, although not optimal, which will be analysed again in future studies.

Given that attitudes have not been evaluated by component and have been analysed by multiple tests (as other authors done previously), a limitation refers to the caution that must be taken in the interpretation of the results presented.

Despite these limitations, this study provides a useful tool that can be a starting point to improve clinical practice respecting the rights of patients.

Future research could include the development of a cross-cultural adaptation of the questionnaire into the English language, as this would allow for international comparisons and to assess how the different cultures can influence in this theme. It could be able to assess the effectiveness of CPR training too.

## Conclusions

CAEPCR questionnaire is the first which successfully linked knowledge and skills of cardiopulmonary resuscitation to the staff’s attitudes towards ethical issues.

The strength of this study is that it delves into an important gap in the scientific literature and provides a tool that allows assessing both the knowledge and the attitude towards the ethical aspects in the cardio respiratory arrest. It could be useful for identify those professional who need a refresher course, and to detect ethical issues in the clinical practice.

In addition health policies should ensure that CPR training is mandatory for healthcare workers, emphasizing ethical aspects.

##  Supplemental Information

10.7717/peerj.6410/supp-1 Appendix S1 The full CAEPCR questionnaire in English language

10.7717/peerj.6410/supp-2 Supplemental Information 1 Database Raw data measurements and codes archive.

## Additional Information and Declarations

### Data Availability

The following information was supplied regarding data availability: Raw data are available in Supplemental Materials.
