# Supplementary material for: Nursing knowledge of and attitude in cardiopulmonary arrest: cross-sectional survey analysis
Source: PeerJ. 2019 Feb 7;7:e6410. doi: 10.7717/peerj.6410 (PMC6368968; doi:10.7717/peerj.6410)
Supplement: Appendix S1 [file peerj-07-6410-s001.docx]

**Sociodemographic data**

**Gender**

Female Male

**Work experience**

<5 years 5-10 years 10-15 years 15-20 years >20 years

**Age**

**Service in which you work**

Medical unit Surgical unit Primary Care Consultation Pediatrics consultation Primary Care Others

**Last time you received a CPR recycling course**

<6 months 6 m- 1year 1-2 years >2 years Never

**Last time he had to attend a cardiac arrest**

<6 months 6 m- 1year 1 -2 years >2 years Never

**How often do you consider it necessary to receive CPR recycling training**

< 6 months 6m-1 year 1 -2 years >2 years Never

**Type of contract**

Eventual Permanent contract

**You feel able to attend a real Cardio Respiratory arrest**

Yes No

**Knowledge questionnaire**

**Given the latest recommendations from the European Resuscitation Council (ERC) 2010 for CPR:**

1. **A family member tells you that a patient is lying on the floor and is not responding, you therefore:**
   1. Raise the alarm
   2. Check whether he/she is breathing
   3. Approach to see whether he/she responds
   4. Initiate CPR (compression/ventilation)
2. **You have checked that the patient is actually unconscious, therefore you now:**
3. Raise the alarm
4. Check whether he/she is breathing
5. Start CPR manoeuvres
6. Re-evaluate regularly
7. **After checking for the absence of breathing and a pulse, you reach the conclusion that the patient is in cardiorespiratory arrest (CRA), therefore you start CPR manoeuvres. Consequently, you must know that the optimal thoracic compression technique comprises:**
8. Compressing the chest at a rate of at least 100 per minute and a depth of at least 5 cm (for adults)
9. Compressing the chest at a rate of at least 60 per minute and a depth of at least 5 cm (for adults)
10. Compressing the chest at a rate of at least 100 per minute and a depth of no more than 4 cm (for adults)
11. Compressing the chest at a rate of no more than 60 per minute and a depth of at least 5 cm (for adults)
12. **According to the ERC 2010 recommendations, the correct compression/ventilation relationship in CPR is**
13. 2:15
14. 15:2
15. 02:30
16. 30:2
17. **The most important changes that have been made in the updates to the CPR guidelines include:**
18. The importance of initiating early uninterrupted thoracic compression.
19. Thoracic compression can be suspended for as long as needed to ensure intubation of the patient.
20. The role of the precordial thump becomes more important.
21. The administration of medicines via the tracheal tube is recommended
22. **During treatment of ventricular fibrillation-related cardiac arrest or pulseless ventricular tachycardia (PVT), 1 mg of adrenaline is administered:**
23. After the second shock and repeat every 3-5 minutes
24. After the third shock and repeat every 3-5 minutes
25. Atropine is administered rather than adrenaline and repeat every 3-5 minutes
26. After the first shock and repeat every 3-5 minutes
27. **For thoracic compression to be of good quality, whenever possible you are recommended to:**
28. Change the person performing the thoracic compression every 4 cycles.
29. The person performing the thoracic compression does not need to be changed if he/she is not tired.
30. Compression can be interrupted briefly to allow the resuscitator to rest.
31. Change the person performing the thoracic compression every 2 minutes, ensuring minimum interruption.
32. **After performing defibrillation you must:**
33. Restart CPR immediately after the shock if defibrillation was unsuccessful, without evaluating heartbeat or taking the pulse.
34. Evaluate the heartbeat and pulse prior to restarting CPR
35. Without evaluating heartbeat or taking the pulse, restart CPR immediately after the shock even if defibrillation was successful.
36. Administer adrenaline prior to restarting CPR.
37. **With regard to the administration route for medicines in CRA, it is true that:**
38. Both venous and intraosteal administration are preferable to the tracheal route for drug administration.
39. Endotracheal administration of adrenaline is preferred if the cause of the CRA was respiratory.
40. An intraosteal needle should be inserted even though the patient has a peripheral line.
41. The route of choice for the administration of adrenaline is endotracheal, therefore intubation of the patient should be prioritised.
42. **The drugs used intravenously must:**
43. Must be followed by at least one bolus of 20 mL fluid
44. Must be followed by at least one bolus of 5 mL fluid
45. Do not need to be followed by a bolus of fluid
46. Only be diluted in physiological saline
47. **What is the most effective treatment for a victim with ventricular fibrillation-related CRA:**
48. IV Adrenaline
49. Precordial thump
50. Defibrillation
51. Intubation

**Attitude questionnaire**

Evaluate on a scale of 1 to 5, where:

| **1**. Strongly disagree 2. Slightly disagree 3. Neither agree nor disagree 4. Slightly agree 5. Strongly agree |
| --- |

Beginning of the questionnaire

|  | | **1** | **2** | **3** | **4** | **5** |
| --- | --- | --- | --- | --- | --- | --- |
| **1** | **Do you consider yourself to be sufficiently well trained to perform CPR** |  |  |  |  |  |
| **2** | **Do you consider that you understand the action protocol for performing CPR in your work area** |  |  |  |  |  |
| **3** | **Do you consider yourself to be personally responsible for being able to perform CPR** |  |  |  |  |  |
| **4** | **Do you consider it to be the responsibility of your work centre to provide you with training to perform CPR** |  |  |  |  |  |
| **5** | **Do you consider that the person with the greatest understanding and experience in the team should be the person to LEAD CPR irrespective of whether they are a physician or a nurse** |  |  |  |  |  |
| **6** | **Do you consider that CPR can be PERFORMED by either physicians or nurses** |  |  |  |  |  |
| **7** | **Do you consider that only healthcare professionals should initiate CPR** |  |  |  |  |  |
| **8** | **Do you consider it appropriate not to start or to interrupt CPR manoeuvres if started when the probability of neurological sequelae is high** |  |  |  |  |  |
| **9** | **Do you consider that the presence of family members does not influence your decision to commence CPR manoeuvres** |  |  |  |  |  |
| **10** | **Do you believe that the information YOU have about the patient may lead you to stop CPR** |  |  |  |  |  |
| **11** | **Do you consider it necessary to identify DNR patients (for example in the hospital or even in the primary care records)** |  |  |  |  |  |
| **12** | **Do you consider it necessary for patients at highest risk of requiring CPR to be identified in the hospital** |  |  |  |  |  |

Thank you for your insights/comment
